# Head and neck squamous cell carcinoma and its correlation with human papillomavirus in people living with HIV: a systematic review

**DOI:** 10.18632/oncotarget.24660

**Published:** 2018-03-30

**Authors:** Manuela Ceccarelli, Emmanuele Venanzi Rullo, Alessio Facciolà, Giordano Madeddu, Bruno Cacopardo, Rosaria Taibi, Francesco D'Aleo, Marilia Rita Pinzone, Isa Picerno, Michele di Rosa, Giuseppa Visalli, Fabrizio Condorelli, Giuseppe Nunnari, Giovanni Francesco Pellicanò

**Affiliations:** ^1^ Department of Clinical and Experimental Medicine, Unit of Infectious Diseases, University of Messina, Messina, Italy; ^2^ Department of Clinical and Experimental Medicine, Unit of Infectious Diseases, University of Sassari, Sassari, Italy; ^3^ Department of Clinical and Experimental Medicine, Unit of Infectious Diseases, University of Catania, Catania, Italy; ^4^ Department of Medical Oncology A, National Cancer Institute of Aviano, Aviano, Italy; ^5^ Department of Pathology and Laboratory Medicine, School of Medicine, University of Pennsylvania, Philadelphia, Pennsylvania, USA; ^6^ Department of Biomedical and Dental Sciences and Morpho Functional Imaging, University of Messina, Messina, Italy; ^7^ Department of Biomedical and Biotechnological Sciences, Human Anatomy and Histology Section, University of Catania, Catania, Italy; ^8^ Department of Pharmacological Sciences, Università del Piemonte Orientale “A. Avogadro”, Novara, Italy; ^9^ Department of Human Pathology of The Adult and The Developmental Age “G. Barresi”, Unit of Infectious Diseases, University of Messina, Messina, Italy

**Keywords:** HNSCC, HIV, head and neck cancer, HPV, papillomavirus

## Abstract

Over the last 20 years we assisted to an increase in the mean age of People Living with HIV and their comorbidities. Especially, there was an increase in Human Papillomavirus-related head and neck squamous cell carcinomas. Despite their increasing incidence in HIV-positive people, mechanisms that lead to their development and progression are only partially understood.

The aim of this review is to identify key data and factors about HPV-related head and neck squamous cell carcinoma in HIV-seropositive patients. Systematic search and review of the relevant literature-peer-reviewed and grey-was conducted using the Preferred Reporting Items for Systematic Reviews and Meta-analysis (PRISMA) guidelines. We included in our review only the 35 full-text articles we considered the most substantial. It is mandatory to improve our knowledge about the interactions existing between HPV and HIV, and about their actions on oral mucosa immune system.

## INTRODUCTION

Over the last 20 years, the so-called post-highly active antiretroviral therapy (post-HAART) era, we assisted to an increase in the mean age of People Living With HIV (PLWH) and comorbidities related to ageing, immune suppression and persistent inflammation [1–65]. Especially, there was an increase in HPV-related oral lesion incidence in HIV-positive persons, including a subset of head and neck squamous cell carcinomas (HNSCC) [66–69]. A higher incidence of tobacco and alcohol use has been reported among HIV-positive persons, along with sexual risk behaviors, occurring in suboptimal immune-status even in the presence of combined Anti-Retroviral Therapy (cART). Therefore, even though in the past it was hypothesized that Highly Active Anti-Retroviral Therapy (HAART), and in particular the use of Protease Inhibitors (PIs), could be accounted for the increase of oral HPV infection and, as a consequence, of HNSCC, it is still not clear if this increase is related to behaviors, to disease factors or to a combination of all them [69–76].

The aim of this review is to identify key data and factors about HPV-related HNSCC in HIV-seropositive patients, and to provide a stepping stone for further studies aimed to clarify the relationship existing between HIV, HPV and HNSCC.

## PHYSIOPATHOLOGY

Despite HNSCCs increasing incidence in PLWH, mechanisms that lead to their development and progression are only partially understood. Substance use and immunosuppression are still under investigation as the variables most frequently associated with their presence [66, 75], even though molecular and epidemiological data confirm the involvement of High-Risk HPV (HR-HPV) in the physiopathology of HNSCC, particularly those localized to the lateral wall of the oropharynx, the base of the tongue and tonsils [77–82]. Other variables, such as irradiation, are currently under investigation [83].

HPV is a DNA virus that can cause lesions anywhere on the cutaneous surface, including the extremities, genitalia and oral mucosa, and establish a persistent oral infection caused by reduced infection clearance and an increased viral load, seen as the presumed precursor for HPV-related oropharyngeal squamous cell carcinoma [81, 84]. However, it cannot establish an infection in healthy skin or mucosa [85]. HPV-DNA can be found in most of HIV/AIDS-associated oral cancer cells, suggesting that HIV can act as a promoter in the onset of HNSCC, disrupting oral mucosa cells' immune functions [76]. As a matter of fact, HIV protein *tat* activates a pathway linked to NF-κB, involving pro-inflammatory cytokines IL-12, IL-6, IL-8 and TNF-α which, when secreted in HIV-infected intraepithelial macrophages and dendritic cells induce apoptosis and disruption of their barrier function [85]. A high concentration of IL-12, IL-6, IL-8 and TNF-α can be found in saliva of PLWH with malignant or pre-malignant oral lesions [76]. NF-κB also upregulates HPV proteins E6 and E7, stimulating their continuous transcription, thus facilitating the progression of HPV-related neoplasms through the inactivation of *p53* and *pRB* [72, 85, 86]. Likewise, HIV-*tat* activates HPV replication, its proteins' expression and the proliferation of HPV-infected keratinocytes. Other HIV proteins, such as *Rev* and *Vpr*, enhance HPV oncogenic activity, stimulating L1 overexpression in epithelial cells or inducing a cell cycle arrest [85].

Moreover, a recent study by Walline et al demonstrated that HPV-related HNSCC express a higher title of the oncoprotein p16 and a lower title of the oncoproteins pRB and p53, even though oncoprotein p53 could be mutant in some subsets of HPV-related HNSCC, usually more aggressive than those expressing a wild type p53 oncoprotein [87].

It appears clear that further studies are required to understand the pathogenesis of HPV-related HNSCC in PLWH.

## RISK FACTORS

The incidence of the HNSCC, particularly those associated to oral HPV infection, has been increasing in people living with HIV in the last 20 years. HPV-related HNSCC have been found to have a Standardized Incidence Ratio (SIR) of 3.2 (95% CI = 2.5-3.4), significantly higher than the general population [79]. HIV-infected persons have, as a matter of fact, a 2-3-fold higher prevalence of oral HPV-infection, compared to the general population, with a highly-variable frequency of high-risk serotypes [67, 84]. Likewise, the incidence of HPV-related HNSCC in PLWH is 2 to 6 times higher than the one in the general population [75, 79, 88, 89].

In this section, we report on the potential risk factors associated to this increase.

### Age

During the pre-HAART era, the incidence of HNSCC increased for males aged 40-64 years, while decreasing for males aged 65 years or older [75]. After the introduction of HAART, it seems that this trend has been diverted. As a matter of fact, studies by Beachler et al [68], and Anaya-Saveedra et al [90], demonstrated that the risk for oral HPV infection, HPV oral lesions (HPV-OLs) and HPV-related HNSCC increases with age. This diverted trend, with an HNSCC incidence increasing with age, had also been observed by Chaturvedi et al in 2009 [91], when they associated the prolonged survival of HIV-infected individuals to the increased incidence of some neoplasms, and by Kreimer et al [82, 92] in two distinct works establishing the incidence and risk factors of HPV-OLs and multiple HPV-seropositivity. On the other hand, a study by Ciarrocca et al [81] demonstrated that HPV-related HNSCC are associated with younger age. Further studies are needed to understand the role of age in the onset of HNSCC.

### Tobacco and alcohol use

PLWH have increased exposure to tobacco and alcohol, which are major risk factors for both the HPV-related HNSCC and the HPV-unrelated HNSCC, compared to the HIV-negative populations [69, 76, 79]. It is known that cigarette smokers have a higher oral HPV prevalence, maybe due to a reduced clearance of the virus, or to an increased HPV viral replication and, as a consequence, a higher viral load, as PLWH have [66, 68, 75, 81, 93]. A reduced clearance of the HPV because of tobacco smoking and alcohol use could be one of the reasons why PLWH have a higher incidence of HNSCC. Moreover, tobacco smoking and alcohol are known agents of oral epithelial damage with a continuous pro-inflammatory activity, which is enhanced by the presence of HIV. Thus, the disruption of the mucosal barrier caused by these agents contribute to the establishment of a chronic HPV infection [73, 85].

### Sexual behavior

As HPV-related HNSCC are caused by the presence of HPV in the oral mucosa, the behaviors that enable HPV to reach the oral cavity and infect the mucosa have to be considered risk factors for HNSCC. An high number of sexual oral partners, as in active fellatio, cunnilingus or rimming, recently or in lifetime, is associated with an high risk of oral HPV infection [68, 75, 81, 82, 92–94]. Moreover, Syrjänen et al [85] demonstrated that PLWH have an higher incidence of high-risk HPV (HR-HPV) serotypes. Curiously, a 2013 work by Beachler et al [89] demonstrated that heterosexual men, and not MSM, like previously affirmed by Del Mistro et al [95], are the population with the higher risk of being persistently positive to an oral HPV infection [79, 89, 95]. However, it is still unclear how and in what measure an oral HPV infection by an HR-HPV serotype causes an increased incidence of HNSCC [69].

### Immunosuppression

A decrease in the number of CD4+ T lymphocytes increases the risk of having an oral HPV infection and developing malignancy [68, 71, 78, 79, 82, 91, 92, 94], while the number of CD8+ T lymphocytes does not seem to be associated with the incidence of HPV-related HNSCC [79]. Oral cancer in PLWH and lower CD4+ T lymphocyte counts have a high mortality rate [75]. It is hypothesized that a low CD4+ T-cell count, even a CD4+ T-cell count around 300 cells/μL, and even around 350 cells/μL in some cases, increases the risk of progression to HNSCC partially because reactivation or reacquisition of oral HPV infection, more than a reduced clearance [68, 69, 87, 89]. It is supposed, though, that a low number of CD4+ T lymphocytes influences the risk of developing an HNSCC early in the carcinogenesis [79]. Despite the collective agreement, further studies are needed to confirm that the CD4+ T lymphocyte count significantly influences the incidence and prevalence of HPV-related HNSCC and how and when it operates.

### Combined anti-retroviral therapy or highly active anti-retroviral therapy (cART or HAART)

PLWH, and even in those currently assuming cART, have an increased risk to have an oral HPV-infection and, as a consequence, an HPV-related malignancy [90]. It is supposed that this risk steadily increased in the post-HAART era because of a prolonged life expectancy: before the introduction of HAART, PLWH developed AIDS and died before the appearance of HPV-related HNSCC [76, 78]. Even though HAART helps controlling the HIV infection, it seems to not reduce the risk of having an oral HPV infection or developing a HPV-related lesion [76, 91, 96]. On the contrary, it could even reduce the barrier function of the oral epithelium, increasing the invasiveness, and thus the malignancy, of the HPV-infection [76]. To support this hypothesis, Anaya-Saavedra et al [90] demonstrated in 2013 that PLWH with an undetectable viral load had a sixfold risk of presenting HPV-OLs. It is still not clear, though, why the introduction of HAART reduced the incidence of AIDS-defining neoplasms associated with oncogenic viruses (Human Herpes Virus 8, HHV8; Epstein-Barr Virus, EBV), but had little to none impact on the incidence of HPV-related tumors, in particular HNSCC [80]. New studies are indeed needed to clarify the influence cART has on the development of oral HPV infections and HPV-related HNSCC.

### HPV genotypes

HPV genotypes are divided into two categories, commonly referred to as HR- HPV and Low-Risk HPV (LR-HPV), to identify their ability of causing cancer. There is evidence that, similarly to cervical cancer, but not to anal cancer, the most common HPV genotype found in HNSCC is HPV-16 [94]. Other genotypes frequently involved in HNSCC in PLWH are -18, -31, -40, -51, -55, -83 [70, 97, 98]. Moreover, HIV-infected persons have not only a higher risk for oral HPV infection, but also for a multiple genotype infection, [76], as well as infection with multiple HR-HPV genotypes regardless of recent or past oral sex [66, 75, 89]. It is important to notice though, that the oral HPV-infection is a causal factor in HNSCC, but it does not work alone, as the inability to clear the infection could have a role in developing a neoplasia [69, 70, 72, 76, 85].

## PRIMARY PROPHYLAXIS (VACCINATION)

Three kinds of vaccine against HPV are available: the quadrivalent Gardasil, against HPV-serotypes 6, 11, 16 and 18; the nine-valent Gardasil-9, against HPV-serotypes 6, 11, 16, 18, 31, 33, 45, 52 and 58; and the bi-valent Cervarix, against HR-HPV-serotypes 16 and 18. Even though it is supposed that HPV vaccination can potentially prevent oral HPV infection as well as cervical and anal ones, there are currently no available data about whether a program of vaccination could decrease the incidence of HNSCC [66, 94]. Nonetheless, it is imperative to improve HPV vaccination rates, not only among young girls, but also and especially among young and HIV-infected people, so that the incidence of HPV-associated cancers—anogenital and oral ones—could be reduced [66, 72, 77].

## SECONDARY PROPHYLAXIS (SCREENING)

It appears clear how it could be necessary to develop a screening test for HPV-related HNSCC. The two main questions are: what should be the technique employed and who should be the receiving end of the screening?

Some authors suggested that cancer prevention strategies should include patient education and clinical vigilance, beginning with visual assessment and digital palpation of the oral-pharyngeal cavity [75, 96]. Some authors recommend the use of oral rinses or saliva to determine the presence of a HPV infection, as there is a strong association with HNSCC and a higher sensibility was demonstrated when compared to other sampling techniques [70, 72, 81, 93]. However, oral rinses cannot establish where the infection is localized, while other sampling techniques like brushings and biopsies could offer these data [93].

Seen that they have a higher HPV-infection incidence, PLWH, and above all those who are affected with anal cancer, should be screened and monitored for HPV-related HNSCC [77]. On the other hand, Beachler et al [79] observed that PLWH's risk of developing an HPV-related HNSCC is only three-fold higher than the general population's, thus a mass-screening policy could not be cost-effective.

Further studies are needed to understand who needs to undergo the screening, and what is the best—the cheapest, with the highest sensitivity—technique to use.

## OUTCOMES

Little is known about the natural history and the treatment outcomes of HPV-related HNSCCs in PLWH [75]. Even though HNSCCs in PLWH are usually more frequent in younger patients, HPV-related ones tend to be usually found in older people and at an advanced stage of both HIV-infection and cancer [69, 76, 79, 88]. Thus, some authors found they have poorer survival rates (57% survival rate at 1 year, and 32% at 2 years) [76, 81, 88]. Moreover, HPV infections in immunocompromised patients are recurrent and recalcitrant to therapy [75]. On the other hand, other authors reported that HPV-related HNSCCs seem to have higher survival rates than HPV-unrelated ones, and that HIV status do not affect the outcome [81, 94]. It seems that p53 expression could be used as a prognostic biomarker for HNSCC-affected PLWH: those with the lowest expression have the best survival, while those with the highest expression have the poorest survival [87].

## MATERIALS AND METHODS

**Figure 1:**
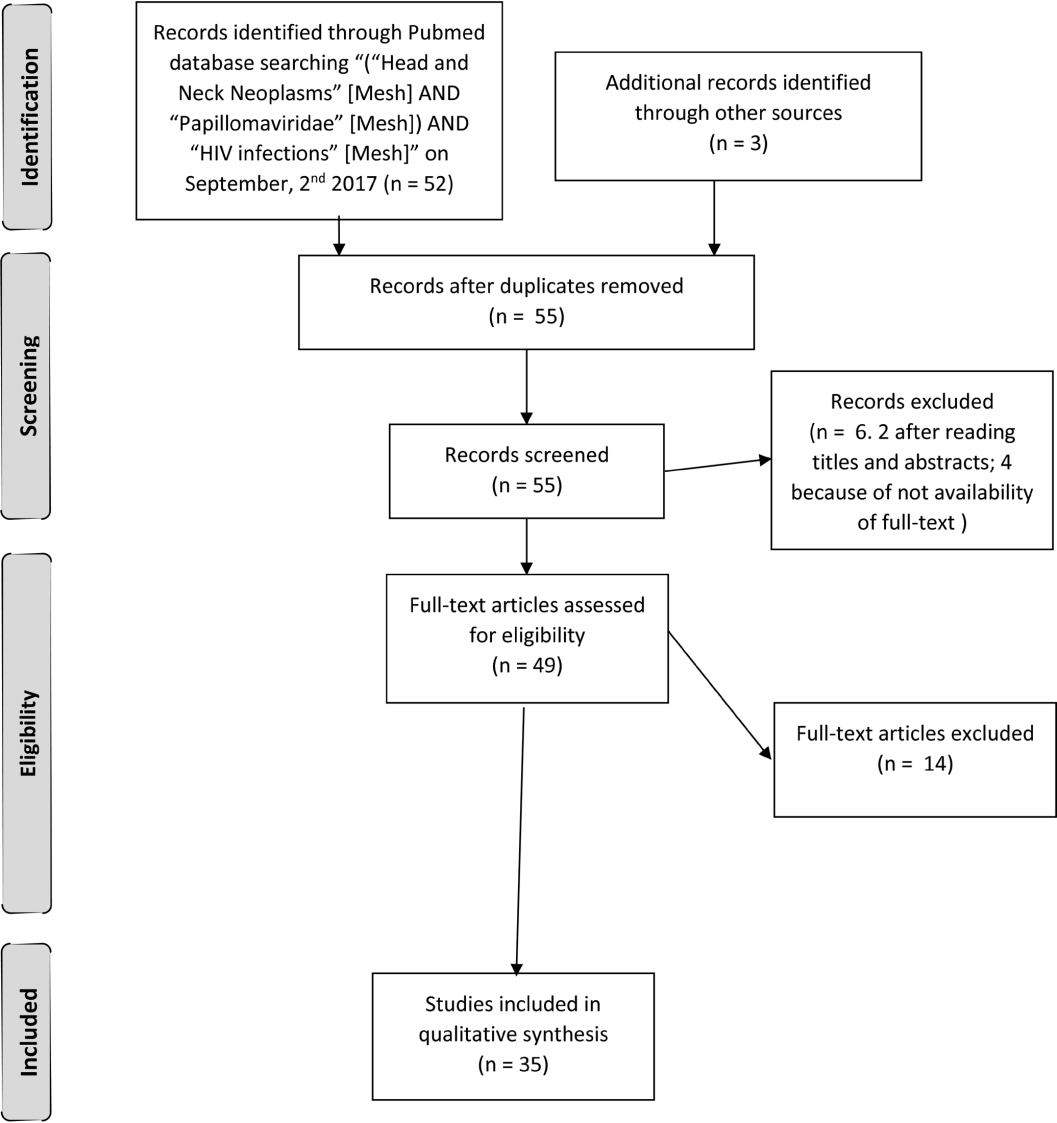
Article assessment diagram

On September 2^nd^, 2017, we performed a MeSH systematic review of the literature to identify the link existing between Head and Neck Cancer (HNC), Human Papilloma Virus (HPV) infection and Human Immunodeficiency Virus (HIV) infection. We searched PubMed applying “(“Head and Neck Neoplasms” [Mesh] AND “Papillomaviridae” [Mesh]) AND “HIV infections” [Mesh]”. We also reviewed the articles references. We limited the inclusion only to articles written in English, published within the last two decades. We identified 55 records and screened them. We excluded 2 articles by title and abstract, because they reported about only one or two queries out of three. Every author independently assessed 49 full-text articles for eligibility. At the end of the assessment we included in our review the 34 full-text articles reporting about HIV-seropositive persons affected by HPV-related HNC we considered the most substantial [66–100] (Figure 1).

## CONCLUSIONS

Despite their current low incidence, HPV-related HNSCC frequency will increase in future, due to the high incidence of HPV oral infection in HIV-infected people. Thus, it is mandatory to improve our knowledge about the interactions existing between HPV and HIV, and about their actions on oral mucosa immune system. Moreover, it is essential to acquire new data about the effects of the vaccination campaign over the incidence of HNSCC. In addition to this, it is required to develop an efficient screening test to determine whether an HPV infection could potentially transform in a malignant lesion. Further studies are needed to achieve these outcomes.

## Abbreviations

HIV: Human Immunodeficiency Virus
PLWH: People Living with HIV
HPV: Human Papillomavirus
HNSCC: Head and neck squamous cell carcinomas
PRISMA: Preferred Reporting Items for Systematic Reviews and Meta-analysis
cART: combined Anti-Retroviral Therapy
HAART: Highly Active Anti-Retroviral Therapy
PIs: Protease Inhibitors
HNC: Head and Neck Cancer
HR-HPV: High-Risk HPV
DNA: Deoxyribose Nucleic Acid
NF-κB: Nuclear Factor kappa-light-chain-enhancer of activated B cells
AIDS: Acquired Immune-Deficiency Syndrome
IL: Inter-Leukin
p53: tumor protein p53
pRB: Retino-Blastoma protein
tat: Trans-Activator of Transcription
rev: Regulatory of Expression of the Viral genoma
vpr: Viral Protein R
p16: tumor protein p16
HPV-OLs: HPV oral lesions
HHV8: Human Herpes Virus 8
EBV: Epstein-Barr Virus
LR-HPV: Low-Risk HPV
MSM: men who have sex with men
